# Seroepidemiological Evidence of Subtype H3N8 Influenza Virus Infection among Pet Dogs in China

**DOI:** 10.1371/journal.pone.0159106

**Published:** 2016-07-14

**Authors:** Pei Zhou, San Huang, Weijie Zeng, Xin Zhang, Lifang Wang, Xinliang Fu, Shoujun Li

**Affiliations:** 1 College of Veterinary Medicine, South China Agricultural University, Tianhe District, Guangzhou, Guangdong Province, People’s Republic of China; 2 Key Laboratory of Comprehensive Prevention and Control for Severe Clinical Animal Diseases of Guangdong Province, Guangzhou, Guangdong Province, People’s Republic of China; 3 Guangdong Engineering and Technological Research Center for Pets, Guangzhou, Guangdong Province, People’s Republic of China; Icahn School of Medicine at Mount Sinai, UNITED STATES

## Abstract

The H3N8 virus and the H3N2 virus are the main subtypes of canine influenza virus (CIV). H3N8 CIV mainly circulates in America, and H3N2 CIV mainly circulates in Asia. However, there was an outbreak of the Asian H3N2 virus in the United States (US) in 2015. Thus, it is important to evaluate the presence of subtype H3N8 virus in dogs in China. From May 2015 to November 2015, 600 sera from pet dogs were collected from Guangzhou, Shanghai, Beijing and Shenzhen for hemagglutination inhibition (HI) assays and microneutralization (MN) assays. Fifty-two (8.66%) of the 600 sera were positive for the subtype H3N2 virus, which matched the previous reports. Five (0.83%) of 600 sera were positive for the subtype H3N8 virus (H3N8 EIV or H3N8 AIV or H3N8 CIV), which is the first report of subtype H3N8 virus infection among dogs in China and remind us to play more attention to this subtype virus. Therefore, further serological and virological surveillance of influenza virus infection among dogs in China is imperative.

## Introduction

Under most circumstances, there are species barriers that hamper interspecies transmission of influenza viruses. However, evolution can help viruses surmount species barriers to sustain transmission in a new host species [1]. Recently, influenza A virus has been shown to infect various hosts, from birds to mammals, and to have varying degrees of adaptation in different hosts [2].

The research history of CIV is relatively short because dogs were long regarded as unsusceptible to influenza viruses. This perception did not change until H3N8 CIV was first identified in the US from what was known as an equine-origin H3N8 influenza virus in January 2004 [3]. The persistence of this subtype H3N8 virus in dogs suggests that the virus has become enzootic in the US [3, 4]. In 2008, the avian-origin H3N2 CIV was first isolated in South Korea [5], and this subtype H3N2 virus was later reported in China [6]. Since then, in China, epidemiological studies of dogs have focused on the subtype H3N2 virus [7–11] and subtype H1N1, H5N1, H7N9, H10N8 [12–16] viruses, which have public health significance. H3N8 CIV had mainly circulated in America, and H3N2 CIV had mainly circulated in Asia. However, this changed during the outbreak of H3N2 CIV in Chicago, and the virus then rapidly spread to numerous states in the US in 2015 [17]. Therefore, it remains possible that H3N8 CIV infection has spread among dogs to reach China or that H3N8 EIV or H3N8 AIV has surmounted species barriers to sustain transmission among dogs. To examine this possibility, we conducted serological surveillance from May 2015 to November 2015 in Guangzhou, Shanghai, Beijing, and Shenzhen, which are the four biggest international cities in China, to evaluate whether the subtype H3N8 virus has infected dogs in China.

## Materials and Methods

### Sample collection, viral antigens, and sera

From May 2015 to November 2015, sera from 600 pet dogs (150 specimens per city) were collected for serology from animal hospitals in Guangzhou, Shanghai, Beijing and Shenzhen and were preserved at -80°C for future testing. The dogs’ characteristics were recorded by the research staff. The samples were tested for EIV-H3N8: A/equine/Heilongjiang/SS1/2013 (H3N8); AIV-H3N8: A/avian/Guangdong/J/2012 (H3N8); CIV-H3N2: A/canine/Guangdong/01/2014(H3N2). Negative control serum was collected from an influenza-negative dog whose serum did not contain antibody against H3N2, H3N8, H9N2, or H1N1 as indicated by HI tests. Positive control sera were prepared from immune rabbits using inactivated viruses. These viruses and control sera were obtained from the Key Laboratory of Comprehensive Prevention and Control for Severe Clinical Animal Diseases of Guangdong Province, the College of Veterinary Medicine, South China Agricultural University.

### Detection of influenza virus antibodies

We used a WHO-recommended HI assay [18]. Briefly, the sera were treated with a receptor-destroying enzyme (RDE, *Denka Seiken 340016 (370013)*) and absorbed with erythrocytes to remove nonspecific inhibitors before the tests. The sera were further diluted to a 1:10 dilution. The samples were two-fold serially diluted in 96-well V bottom microtiter plates, and 4 hemagglutination units (HAU) of the virus were added to each well. The sera and virus mixtures were incubated at room temperature for 30 min. Then, 1% red blood was added to all wells. The plates were incubated at room temperature and read after 30 min. The serum titer was expressed as the reciprocal of the highest dilution of serum at which hemagglutination was inhibited. All assays were conducted twice with triplicate wells each time, and the final titer was only accepted when both replicates yielded matching results.

Sera from dogs with an HI titer ≥ 20 were confirmed with MN recommended by the WHO [18]. Briefly, the sera were treated with RDE, and two-fold serial dilutions were performed in 96-well polystyrene immunoassay plates (Nunclon Delta surface, Nunc, Denmark). Then, equal volumes of virus diluent containing influenza virus at 100 TCID_50_/50 μl were mixed with the diluted sera. After incubation for an hour, 1.5×10^4^ MDCK cells were added to each well. After incubation for 18–22 hours, the monolayers of MDCK cells were washed with PBS and fixed in cold 80% acetone for 10 minutes. Finally, the viral nucleoprotein (NP) was detected by enzyme-linked immunosorbent assay (ELISA, Immune Technology Company). An HI titer ≥ 20 and an MN titer ≥ 80 is considered positive evidence of previous H3N8 virus infection. Additionally, the specimens with an HI titer ≥ 20 against H3N8 virus were further evaluated for human H3N2 influenza virus (HuIV) antibody using an enzyme-linked immunosorbent assay (ELISA, *H3N2 HA1 Hemagglutinin*, *HA1 ELISA Kit*, *Prod*. *No*.: *DEIA252*, *Creative Diagnostics company*) according to the manufacturer’s instructions.

## Results

After the outbreak of H3N2 CIV in Chicago, US, in April 2015, we sought to evaluate whether subtype H3N8 virus infection had occurred among dogs in China. Thus, from May 2015 to November 2015, we collected sera from 600 pet dogs in Guangzhou, Shanghai, Beijing, and Shenzhen. In total, using a cutoff of HI ≥1:20, 52 (8.66%) of the 600 sera were positive for canine H3N2 virus, 8 (1.33%) of the 600 sera were positive for equine H3N8 virus, and 4 (0.67%) of the 600 sera were positive for avian H3N8 virus (Table 1). Furthermore, the MN assay was conducted to confirm the presence of equine H3N8 and avian H3N8 viruses antibody in the sera (HI ≥1:20). Ultimately, using cutoffs of HI ≥1:20 and MN ≥1:80, 5 specimens were positive for equine H3N8 virus, and 3 specimens were positive for avian H3N8 virus which is also positive for equine H3N8 (Table 2). One of these five H3N8 positive specimens had both subtype H3N2 virus antibody and subtype H3N8 virus antibody (Table 2). Additionally, HI assays were conducted to evaluate the cross-reactivity between these viruses. There was strong cross-reactivity between H3N8 EIV and H3N8 AIV, and no cross-reactivity was observed between H3N8 EIV or H3N8 AIV and H3N2 CIV (Table 3). Additionally, ELISA assays were conducted to eliminate the possibility of H3N2 HuIV cross-reactivity for the subtype H3N8 virus (data not shown).

**Table 1:** Prevalence of elevated antibody titers against the subtype H3N2 and H3N8 viruses among dogs by HI assay, China, 2015.

Cities	No.	CIV-H3N2 HI seroprevalence						No. of HI ≥1:20 (%)	EIV-H3N8 HI seroprevalence						No. of HI ≥1:20 (%)	AIV-H3N8 HI seroprevalence						No. of HI ≥1:20 (%)
		1:20	1:40	1:80	1:160	1:320	1:640		1:20	1:40	1:80	1:160	1:320	1:640		1:20	1:40	1:80	1:160	1:320	1:640	
GZ	150	3	5	3	3	1	1	16(10.67)	1	0	1	0	0	0	2(1.33)	1	0	0	0	0	0	1(0.67)
SH	150	4	3	3	2	2	0	10(6.67)	0	1	0	1	0	0	2(1.33)	0	1	0	0	0	0	1(0.67)
BJ	150	2	1	7	1	3	0	14(9.33)	1	0	2	0	0	0	3(2.00)	0	2	0	0	0	0	2(1.33)
SZ	150	3	2	4	0	2	1	12(8.00)	0	1	0	0	0	0	1(0.67)	0	0	0	0	0	0	0(0.00)
Total	600	12	11	17	6	8	2	52(8.66)	2	2	3	1	0	0	8(1.33)	1	3	0	0	0	0	4(0.67)

Note: GZ: Guangzhou; SH: Shanghai; BJ: Beijing; SZ: Shenzhen

EIV-H3N8: A/equine/Heilongjiang/SS1/2013(H3N8); AIV-H3N8: A/avian/Guangdong/J/2012(H3N8)

CIV-H3N2: A/canine/Guangdong/01/2014(H3N2).

**Table 2:** Characteristics of the study subjects whose sera were reactive against subtype H3N8 viruses.

Number	Age	Traveled^a^	Influenza vaccinated	Collect city	Collect day	Influenza-like symptoms	EIV-H3N8		AIV-H3N8		CIV-H3N2	
							Titer by HI	Titer by MN	Titer by HI	Titer by MN	Titer by HI	Titer by MN
1	7 years	No	No	Guangzhou	2015.6.04	No	1:20	1:20	0	0	0	0
2	2 months	No	No	Guangzhou	2015.6.04	No	**1:80**^b^	**1:160**	**1:20**	**1:80**	0	0
3	3 months	No	No	Shanghai	2015.7.13	No	1:40	0	0	0	1:20	0
4	9 months	No	No	Shanghai	2015.7.13	No	**1:160**	**1:320**	**1:40**	**1:80**	0	0
5	4 years	No	No	Beijing	2015.7.24	No	1:20	0	0	0	0	0
6	3 years	No	No	Beijing	2015.7.24	No	**1:80**	**1:160**	**1:40**	**1:80**	0	0
7	2 years	No	No	Beijing	2015.6.24	No	**1:80**	**1:80**	1:40	1:40	**1:40**	**1:80**
8	5 months	No	No	Shenzhen	2015.10.07	No	**1:40**	**1:80**	0	0	0	0

Note: EIV-H3N8, A/equine/Heilongjiang/SS1/2013(H3N8); AIV-H3N8, A/avian/Guangdong/J/2012(H3N8); CIV-H3N2, A/canine/Guangdong/01/2014(H3N2).

^a^ include dogs and their house mates.

^b^ Bold indicates positive specimen by HI titer ≥ 20 and MN titer ≥ 80.

**Table 3:** HI titers of control sera against reference virus strains.

Reference strains	Positive sera against			
	EIV-H3N8	AIV-H3N8	CIV-H3N2	Negative serum
EIV-H3N8	>1280	640	**/**	**/**
AIV-H3N8	1280	>1280	**/**	**/**
CIV-H3N2	**/**	**/**	>1280	**/**

Note: EIV-H3N8, A/equine/Heilongjiang/SS1/2013(H3N8)

AIV-H3N8, A/avian/Guangdong/J/2012(H3N8)

CIV-H3N2, A/canine/Guangdong/01/2014(H3N2).

Positive sera were prepared from immune rabbits using inactivated viruses. / indicates HI titers≤10.

## Discussion

Since CIV was first identified in the US, the H3N8 CIV has mainly been circulating in America, and H3N2 CIV has mainly been circulating in Asia [3, 5, 6]. However, there was an outbreak of the Asian H3N2 CIV in the US in 2015 [17]. Hence, it is urgent to investigate whether there have been previous subtype H3N8 virus infections among dogs in China. Through serological surveillance from May 2015 to November 2015 in Guangzhou, Shanghai, Beijing and Shenzhen, we found that 5 of 600 pet dogs were previously infected with subtype H3N8 virus (H3N8 EIV or H3N8 AIV or H3N8 CIV). Our results were consistent with previous reports, with an 8.66% prevalence of subtype H3N2 virus infection among dogs [7, 11].

H3N8 EIV was first reported to surmount species barriers to cause sustained transmission among dogs in the US in 2004 [3, 4]. Subsequently, this transmission of this virus from horses to dogs appeared in Australia in 2007 [19, 20]. In 1993, H3N8 EIV was transmitted to China [21], and a previous study reported that H3N8 EIV infected pigs and donkeys in China [22, 23]. Therefore, it is possible that this virus crossed species barriers to infect directly to dogs in China. In addition, because Asian H3N2 CIV recently transmitted to US [17], it seems that there are potential transmission routes for CIV between America and Asian countries, including pet dog transportation, army dog introduction to the country, and dog rescue. H3N8 AIV is one of the most common subtype in wild birds [24] and is low pathogenic avian influenza virus (LPAV) for birds. Significantly, H3N8 AIV has established lineages in horses [25] and then transmit to dogs [3], and also have crossed species barriers to infect seals [26]. This suggests that H3N8 AIV could cross species barriers to infect mammals. According to Table 2, these positive sera dogs are no influenza-like symptoms and have no travel (including their house mates), it seems logical that this is the domestic transmission event in China. It is possible that H3N8 AIV crossed species barriers to infect dogs in China, like H3N2 AIV.

Here, we first reported the seroepidemiological evidence of subtype H3N8 virus infection among dogs in China. Further and continuous surveillance needs to be performed for this subtype virus among dogs in China. Currently, dogs are infected with different subtypes of influenza viruses, such as H3N8 [3], H3N2 [6], H1N1 [27], H3N1 [16], H9N2 [28], H5N2 [29] and H5N1 [30], and previous studies have reported that it is possible for dogs to become a “mixing vessel”, similar to pigs [31–33]. Therefore, continuous serological and virological surveillance needs to be performed among dogs.

In this study, there are two potential limitations should be kept in mind. First, although no cross-reactivity between subtype H3N8 virus and H3N2 CIV or H3N2 HuIV, the strong cross-reactivity between H3N8 EIV and H3N8 AIV can not be eliminated. Nonetheless, the subtype H3N8 virus infects in dogs is been verified in this study. Second, no H3N8 CIV that is known as an equine-origin was used in this study to testify the results, but the origin H3N8 EIV was used as a substitute.

### Ethical approval

This study protocol was reviewed and approved by the Institutional Review Board of South China Agricultural University.
